# Comparison of operative microscope and exoscope for execution of microanastomoses on an artificial model

**DOI:** 10.3389/fsurg.2025.1573333

**Published:** 2025-08-08

**Authors:** T. Calloni, G. Carone, M. Cavaliere, C. de Laurentis, M. Bussa, L. Antolini, F. Nicolosi, G. G. Carrabba, M. M. Fontanella, M. Cenzato, F. DiMeco, F. Acerbi, C. G. Giussani

**Affiliations:** ^1^ Department of Neurosurgery, IRCCS San Gerardo dei Tintori, Monza, Italy; ^2^ School of Medicine and Surgery, University of Milano- Bicocca, Milan, Italy; ^3^ Department of Neurosurgery, IRCCS Carlo Besta Neurological Institute Foundation, Milan, Italy; ^4^ Department of Neurosurgery, ASST Fatebenefratelli—Sacco, Milan, Italy; ^5^ Department of Neurosurgery, Groupement Hospitalier Est, Hospices Civils de Lyon, Lyon, France; ^6^ Faculté de Médecine Lyon Est, Claude Bernard University Lyon 1, Lyon, France; ^7^ Division of Neurosurgery, Department of Medical and Surgical Specialties, Radiological Sciences and Public Health, University of Brescia, Brecia, Italy; ^8^ Department of Neurosurgery, Great Metropolitan Hospital Niguarda, Milan, Italy; ^9^ Department of Pathophysiology and Transplantation, University of Milan, Milan, Italy; ^10^ Department of Neurosurgery, Johns Hopkins Medical School, Baltimore, MD, United States; ^11^ Department of Translational Research and New Technologies in Medicine and Surgery, University of Pisa, Pisa, Italy; ^12^ Neurosurgery Department, Azienda Ospedaliero-Universitaria Pisana, Pisa, Italy

**Keywords:** microanastomoses, microvascular training, education, residents, learning curve, 3D exoscope, microscope, vascular neurosurgery

## Abstract

**Introduction:**

While the improved ergonomics, depth of field, and freedom of movement offered by Exoscopes compared to Operative Microscopes are well established, their value in surgical education and training is often mentioned but remains poorly documented.

**Methods:**

In this study, we used a using a slightly modified version of the NOMAT score to compare the microvascular anastomoses on an artificial model made using traditional Operative Microscopes and the Orbeye 4K 3D Exoscope. Each participant performed the task 3 times.

**Results:**

The results showed that microscope users initially scored higher in several aspects, likely due to greater prior familiarity with the device. However, by the third repetition, the differences were no longer significant, demonstrating that the Exoscope is not inferior to the traditional Microscope in laboratory training. Moreover, the Exoscope group exhibited a faster learning curve for specific skills, highlighting its potential for early adoption by young surgeons.

**Discussion:**

These findings emphasize the educational promise of Exoscopes, particularly in facilitating a smooth transition from traditional microscopes. However, further studies with larger sample sizes and extended training periods are needed to validate these conclusions.

## Introduction

Exoscopes are a relatively recent addition to the range of devices used for intraoperative illumination and visualization. Modern models can deliver 4K 3D video streams from a compact camera to large, high-definition screens. Superior ergonomics (1), uncoupling of surgeon's position from operative angle (2), longer focal distance, ampler field of view (3) are frequently described advantages of exoscopes over traditional operative microscopes (OMs).

Among these advantages, the educational potential of exoscopes has been frequently highlighted (4). The availability of the same high-quality images available to the lead surgeon to all the personnel in the OR is frequently reported as one of the reasons (5, 6).

The advantages of exoscopes in hands-on learning are not obvious: perhaps unsurprisingly, multiple investigators have demonstrated that over time subjects improve in executing tasks with these devices (7–9), a demonstration of the superiority of exoscopes to microscopes in learning of different skills has instead proved elusive (10), even in previous studies by our group (11).

Microvascular anastomoses are challenging (12) and have been extensively used to test skill acquisition using the exoscope (13) and comparing it to the operative microscope (14–18). However, outcomes in these comparisons have been inconsistent, leaving the question of exoscope effectiveness in direct surgical training unresolved.

## Materials and methods

### Volunteers

Twenty residents from the neurosurgery residency programs of three Universities (Università degli Studi di Milano, Università di Milano—Bicocca, Università di Brescia) volunteered to join the study. They answered an enrollment questionnaire concerning previous surgical experience, microvascular experience and attendance of specific courses, experience with different visualization devices**.**

Based on the questionnaire results, the residents were divided into two cohorts. The cohorts were balanced based on the self-reported previous surgical experience. The cohorts were randomly assigned to the exoscope (Orbeye 4K 3D, Olympus, Japan) and the microscope (LEICA OHX, Leica Microsystems, Germany or Pentero, Zeiss, Germany, depending on the available device).

The residents were given a lecture on anastomoses by an expert vascular neurosurgeon (F.A.) including demonstrating a microvascular anastomosis using the same model and instruments they would be later given and reading materials (books and technical notes concerning microvascular anastomoses) all of which made available to participants for review at any time.

### Task

**Figure 1:**
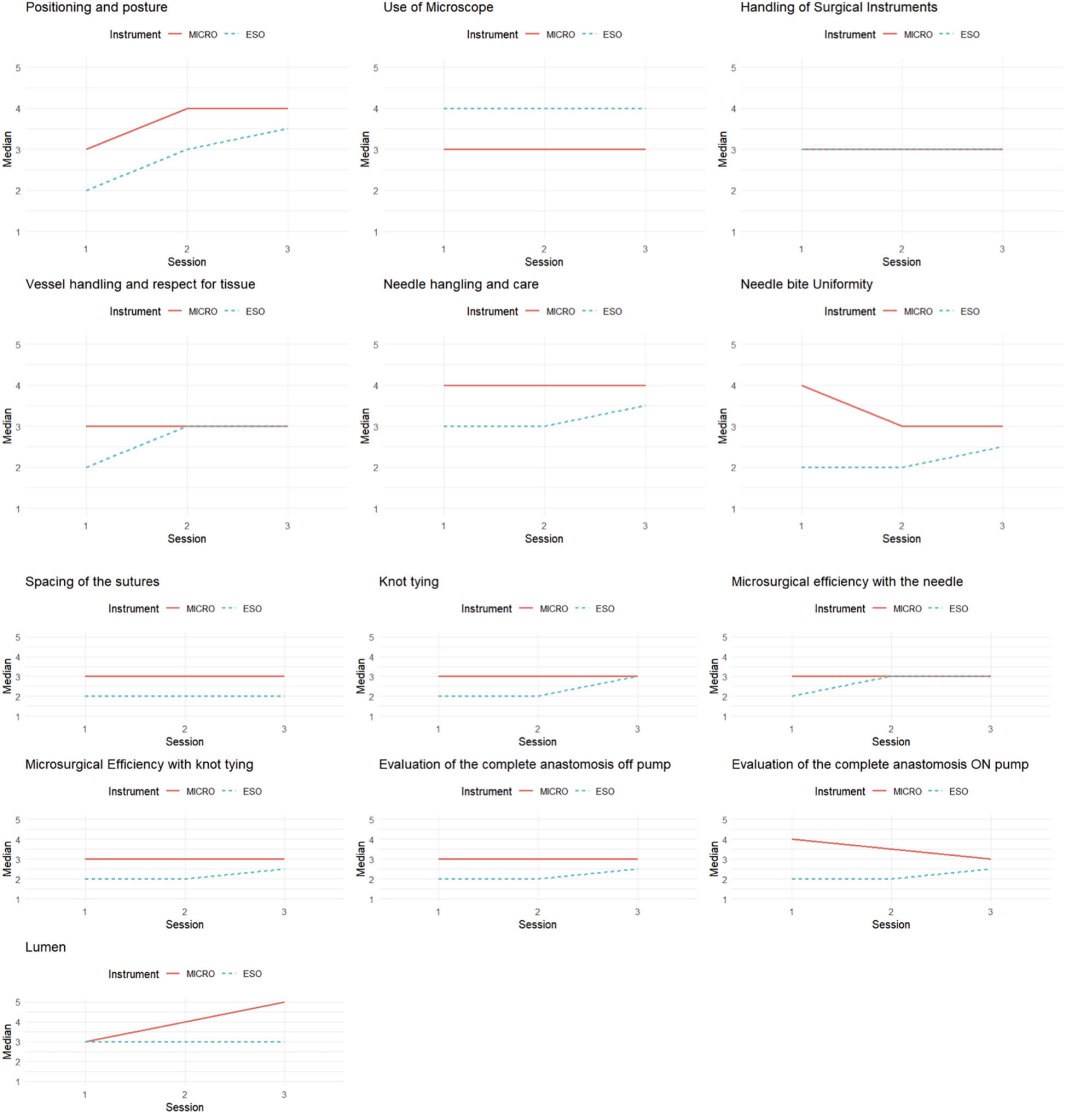
Spaghetti plots describing average scores for each task across 3 repetitions.

Each resident performed the exercise, consisting of an end-to-end microvascular anastomosis on a 2 mm artificial vessel with dissectible adventitia on a simulator (Mycro, UpSurgeon, Italy), with the assigned device three times, each try 14–21 days apart from the previous one. In this model the vessel to be anastomosed is placed on top of a puck of soft material. The simulators were placed on a Mayo table, the residents were sitting (Figure 1). 9/0 surgical sutures were given to the residents. Surgical instruments available were the ones included in the Upsurgeon Mycro set (two forceps, one scissor, one needle holder).

### Evaluation

While multiple paradigms for anastomosis evaluation have been developed and validated, we used a slightly modified version of the Northwestern Objective Microanastomosis Assessment Tool (NOMAT) (19) for this study: the item 3 (Understanding of the surgical instruments) was removed due to the small number of instruments made available to the volunteers.

As required by the NOMAT, procedural videos were recorded and acquired from the instruments and the residents were filmed while performing the task. Per NOMAT, the anastomoses were tested and opened by the investigators in charge of supervising the exercises, this was also recorded.

Due to the complexity of the task, one hour was established as the time limit, the time was not considered in the scoring.

An expert vascular surgeon (F.A.) scored each anastomosis. Blinding to the instrument and the residents' identity was impossible due to some items of the NOMAT score requiring subjects to be filmed while performing the anastomoses, the evaluator was blinded to which repetition of the exercise he was scoring.

Due to the laboratory nature of the study, the use of artificial models and the volunteering of the subjects, ethical board authorization was not required.

### Statistical analysis

**Figure 2:**
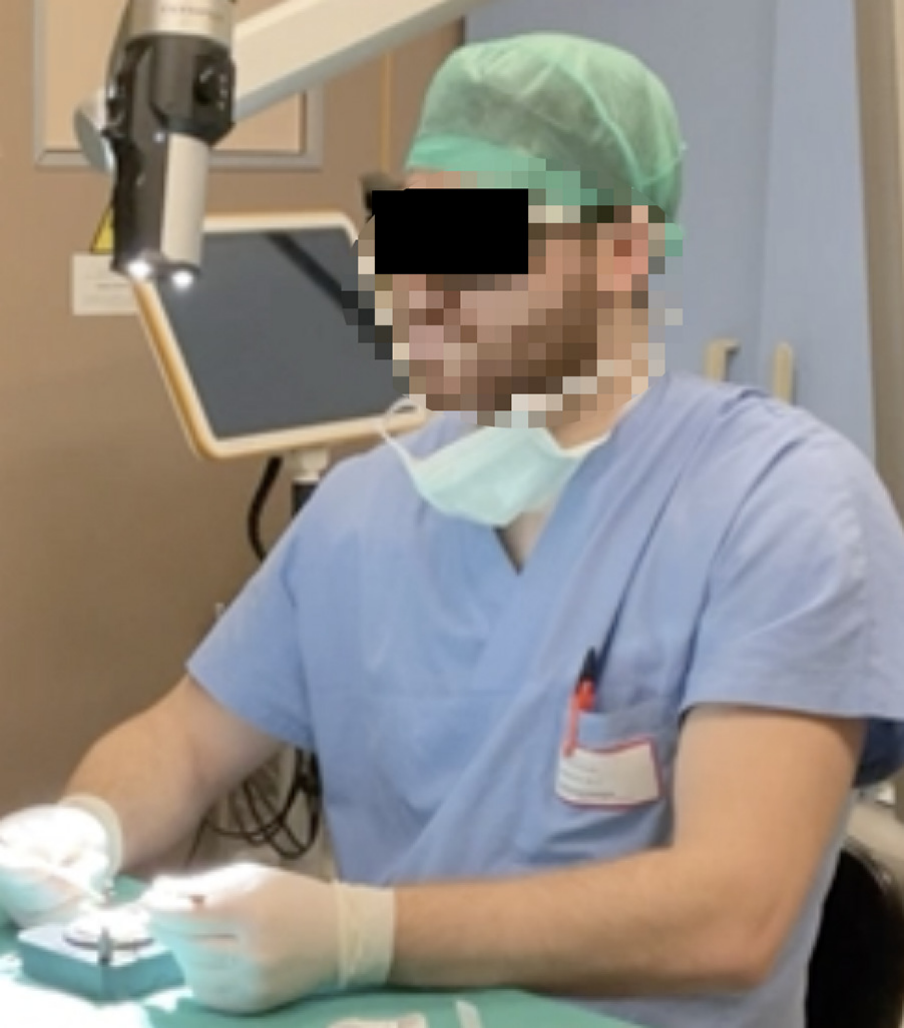
One of the volunteers performing the task with the exoscope.

The statistical analysis began with a descriptive comparative analysis of the groups defined by the instrument used (ESO and MICRO), using the median for the thirteen items of the NOMAT scale. To graphically represent the temporal trends of the variables in the two groups, spaghetti plots (Figure 2) were created, illustrating the medians of the 13 items across the three observation times (T1, T2, T3).

Subsequently, for each item of the NOMAT scale, a mixed-effects model was constructed with the following formula: dependent variable∼Instrument∗Session + (1∣ID).

The models included fixed effects for the group (Instrument), time (Session), and their interaction, while the random intercept effect [(1|ID)] accounted for individual differences between participants. A significance level of 5% was adopted for all analyses. The analysis was performed using R software, version 4.2.3.

## Results

To evaluate how the devices impacted the resident's performance, the scores achieved by the two groups (Exoscope and Microscope) during the first and the third repetition of the task were compared. Results are reported in Table 1. This analysis demonstrated that during the first try, the microscope cohort scored better in Items 1, 6, 7, 8, 9, 10, 11, 12 and 13, and a trend toward statistical significance in items 4 and 5.

**Table 1:** Comparison of results during the first and the third time with each instrument across each of the considered NOMAT items.

Item	Microscope group T1 vs. Exoscope group T1			Microscope group T3 vs. Exoscope group T3		
	Coef.	95% CI	*p*-value	Coef.	95% CI	*p*-value
Operator positioning and posture	−0.79	−1.54; −0.05	0.05	0.583	−0.38; 1.53	0.26
Use of the surgical microscope	0.56	−0.34;1.46	0.25	0.56	−0.61; 1.74	0.37
Handling of surgical instruments	−0.64	−1.28; 0.01	0.07	−0.05	−0.91; 0.82	0.91
Vessel handling and respect for tissue	−0.84	−1.64; −0.03	0.06	0.62	−0.42; 1.68	0.27
Needle handling and care	−0.75	−1.33; −0.17	**0**.**02**	0.42	−0.25; 1.08	0.24
Needle bite uniformity	−1.22	−2.07; −0.37	**0**.**01**	0.92	−0.16; 1.99	0.12
Spacing of the sutures	−1.11	−1.88; −0.34	**0**.**01**	0.28	−.077; 1.32	0.61
Knot tying	−1.26	−1.99; −0.53	**0**.**002**	0.78	−0.03; 1.60	0.07
Microsurgical efficiency with the needle	−1.21	−1.92; −0.51	**0**.**002**	0.75	−0.19; 1.70	0.14
Microsurgical efficiency with knot tying	−1.17	−1.91; −0.42	**0**.**005**	0.79	−0.10; 1.70	0.10
Evaluation of the complete anastomosis off-pump	−1.08	−1.89; −0.27	**0**.**02**	0.45	−0.70; 1.60	0.47
Evaluation of the complete anastomosis on-pump	−1.48	−2.4; −0.57	**0**.**004**	0.65	−0.63; 1.92	0.34
Lumen	−0.29	−1.45; 0.88	0.65	−0.99	−.2.34; 0.34	0.19

Bold: statistically significant items.

Legend: T1 = First time; T3 = Third time.

By the third repetition, no significant difference was observed favoring either instrument, only in Item 9 (Knot tying) a trend toward significance was observed.

We then performed intra-cohort analyses, comparing the scores of the first and last task repetition (Table 2). In the microscope group, a statistically significant difference was found only in item 14 (Lumen). The exoscope cohort demonstrated a significant improvement in Item 1, 6, 7 and 9.

**Table 2:** Comparison of intra-cohort results during the first and the third task repetitions across each of the considered NOMAT items.

Item	Coef.	95% CI	*p*-value	Coef.	95% CI	*p*-value
Operator positioning and posture	0.37	−0.29; 1.03	0.30	0.95	0.24; 1.65	**0** **.** **02**
Use of the surgical microscope	−0.10	−0.65; 0.74	0.81	0.46	−0.31; 1.22	0.26
Handling of surgical instruments	0.31	−0.19; 0.80	0.24	0.21	−0.44; 0.85	0.54
Vessel handling and respect for tissue	0.05	−0.69; 0.78	0.89	0.67	−0.11; 1.47	0.12
Needle handling and care	0.31	−0.18; 0.80	0.24	0.73	0.26; 1.21	**0**.**01**
Needle bite uniformity	−0.03	−.0.80; 0.75	0.95	0.89	0.11; 1.67	**0**.**04**
Spacing of the sutures	0.05	−0.62; 0.74	0.89	0.36	−0.38; 1.10	0.36
Knot tying	0.19	−0.46; 0.83	0.57	0.97	0.44; 1.49	**0**.**002**
Microsurgical efficiency with the needle	−0.12	−0.67; 0.44	0.69	0.65	−0.04; 1.34	0.08
Microsurgical efficiency with knot tying	−0.27	−0.85; 0.29	0.36	0.51	−0.22; 1.25	0.19
Evaluation of the complete anastomosis off-pump	0.20	−0.52; 0.92	0.59	0.65	−0.23; 1.53	0.17
Evaluation of the complete anastomosis on-pump	−0.38	−1.33; 0.57	0.44	0.28	−0.61; 1.17	0.56
Lumen	1.20	0.24; 2.16	**0**.**04**	0.15	−0.87; 1.12	0.78

Bold: statistically significant items.

Legend: T1 = First time; T3 = Third time.

## Discussion

In our study, the residents assigned to the microscope cohort initially performed significantly better than those in the exoscope cohort across 9 of the 14 NOMAT items, with 2 additional items showing a trend toward statistical significance. However, by the third repetition, the difference had substantially vanished, with one item trending toward significance and no item achieving significance. This contrast with prior studies, which describe a superiority for the microscope in microvascular anastomoses (16).

Within-cohort analyses revealed distinct trends: the exoscope cohort demonstrated significant improvement in 4 items by the third repetition, compared to just one item for the microscope cohort. While the volunteers were assigned to each group based on the answers they gave to the pre-study questionnaire, most volunteers reported having more experience with the microscope, some had not used the exoscope previously. This might explain the initial advantage of the microscope group. The faster progression of the exoscope cohort in our study seems thus an effect of a lower starting point, as by the third repetition the two cohorts scored comparably. It could be argued that the exoscope group would have maintained the more rapid learning curve across successive repetitions of the task, and the study was not long enough to document the eventual overtaking, but we believe what we observed the exoscope cohort overcome an initial disadvantage due to most volunteers being unfamiliar with the instrument. This might point toward the possibility for faster switching from microscope to exoscope than described in other studies (7). This might explain the difference in results with respect to other studies describing better performances with the microscope (16): our cohort was made up of residents, not “advanced” nor “highly experienced” microsurgeons, thus the instrument-specific skill gap our cohort needed to close to achieve comparable results across the two instruments was much smaller. An early career switch intuitively seems easier, and our results confirm it.

While distributed practice is the better way to achieve technical proficiency in challenging tasks (12), learning microvascular anastomoses requires more practice than the 3 repetitions in our study (20).

A surprising result was the microscope cohort's higher scores in “Operator Positioning and Posture” (Item 1) during the first task. This appears to be in contrast with the often quoted ergonomics advantage the exoscope has over the microscope (2, 21, 22). In our opinion, the explanation of this discrepancy is twofold: the very user-friendly positioning of the model on a Mayo table, flat and set in the most comfortable way for the user to perform the task without taking into account any of the factors than play into positioning during real-life procedures, while the worse score in the first repetition is a likely effect of the majority of volunteers being unfamiliar with the instrument.

Overall, while the exoscope *per se* does not seem to hinder the acquisition of a new skill by residents and the initial lack of familiarity is rapidly overcome, it doesn't lead to overall better results either, at least in a short amount of time. The frequently described educational value of the exoscope (4) thus seems to mostly derive from better visualization for the observing personnel and allowing better supervision of trainees during *in-vivo* procedures (23).

## Conclusions

This study compared the performance of neurosurgery residents in performing microvascular anastomoses on an artificial model using either a traditional operative microscope or a 4K 3D exoscope. Initially, the microscope cohort demonstrated superior performance, likely due to greater familiarity with the device. However, the exoscope cohort showed a steeper learning curve, quickly closing the performance gap. By the third repetition, there were no significant differences in outcomes between the two groups. These findings indicate that exoscopes are a viable alternative to traditional microscopes, particularly for novice surgeons. The rapid adaptability observed in the exoscope cohort suggests that transitioning to this technology early in training may be both feasible and effective. While exoscopes may not inherently provide superior outcomes in short-term laboratory training, their potential educational advantages, including improved visualization for observers and supervisors, remain promising for real-world applications.

Further research with larger cohorts and extended training periods is needed to fully understand the long-term learning curves and real-world applicability of exoscopes in surgical education.

### Limitations

One major limitation of our study is the relatively small number of subjects in each group and the limited number of repetitions, which limits the study power and ability to generalize conclusions. For the same reason, while we tried to balance the two cohorts based on self-reported previous experience in microscopic and exoscopic surgery, we did not perform subgroup analyses.

We chose to have the volunteers perform an end-to-end anastomosis, to avoid increasing the task complexity (and in keep with the exercise in the NOMAT study). In turn this limits the generalizability of this study to *in vivo* anastomoses and might even have hidden some advantages of either instrument in real life scenarios.

Concerning the evaluation process: blinding of the evaluator to both volunteers' identity and instrument was not possible, as discussed in the text. Furthermore, as a single evaluator was used in this study, conclusions regarding items 1 and 2, which have a low InterRarer Reliability (24), can hardly be generalized.

It is furthermore likely none of the volunteers achieved real-world proficiency in microvascular anastomoses based just on the participation in this study, nor it's possible to speculate where the learning curves would end in a study comprising more repetitions of the task across a longer time.

## Data Availability

The raw data supporting the conclusions of this article will be made available by the authors, without undue reservation.
